# Designing a Model for Developing Food Literacy Among Youth: Insights from Summer Camps

**DOI:** 10.3390/nu18132168

**Published:** 2026-07-03

**Authors:** Laurence Laberee, Sophie Desroches, Karine Chamberland, Mylène Turcotte, Véronique Provencher

**Affiliations:** 1 Centre Nutrition, santé et société (NUTRISS), Institut sur la nutrition et les aliments fonctionnels (INAF), Université Laval, Québec, QC G1V 0A6, Canada; laurence.laberee.1@ulaval.ca (L.L.); sophie.desroches@fsaa.ulaval.ca (S.D.); mylene.turcotte@fsaa.ulaval.ca (M.T.); 2 École de nutrition, Université Laval, Québec, QC G1V 0A6, Canada; 3 Fondation Tremplin Santé, Québec, QC G1H 0A4, Canada; kchamberland@fondationldt.com

**Keywords:** food literacy, nutrition education, summer camps, healthy eating, health promotion, food environment, dietary behaviors, child nutrition

## Abstract

**Background/Objectives:** Although food literacy is extensively studied in schools, the concept is less explored in the context of summer camps, which are also interesting learning environments. This qualitative study aimed to explore the clarity, usefulness and relevance of an adapted food literacy model for summer camps with camp counselors, camp managers and registered dietitians (RDs). **Methods**: Six semi-structured focus groups with counselors (*n* = 28) were conducted at summer camps located in the provinces of Quebec and Ontario, Canada. Semi-structured individual interviews were carried out online with camp managers (*n* = 5) and RDs (*n* = 6). Through an inductive approach, a thematic content analysis of transcribed verbatim was performed. Using a bottom-up approach, second and third final versions of the adapted food literacy model were developed. **Results**: Counselors and managers showed their intention to use an easy-to-use food literacy model. To improve their understanding, they proposed clarifying who will use the model and adding pictures. They expressed the need to be supported by incorporating concrete activity ideas and training to enhance the model’s usefulness. RDs highlighted that the model covered essential themes of food literacy. They expressed the need to clarify certain model components to make them easier to understand and more applicable to youth. **Conclusions**: This study contributed to the co-creation with participants of a clear, useful and relevant food literacy model tailored to the context of summer camps. Such a model will help camp counselors to implement relevant healthy eating actions and multiply opportunities to promote healthy habits among youth.

## 1. Introduction

Over the years, global food systems have evolved, shaped by worldwide drivers of change such as urbanization, population growth and advanced food-processing technologies [1]. These transformative factors have contributed to a nutrition transition, characterized by a progressive shift away from traditional and minimally processed diets toward a higher intake of energy-dense foods, refined grains, added sugars, saturated fats, sodium, and red and processed meats [2]. Such changes in dietary patterns have significant consequences for population health, especially given the well-documented associations between the high consumption of ultra-processed foods, which often present suboptimal nutritional profiles, and increased risks of obesity and chronic diseases [3]. As a result, with such foods widely available, individuals are constantly navigating a complex food environment, which makes it a real challenge for them to adopt healthy eating habits [4]. Thus, developing food literacy represents a way to empower people in making reasoned and informed food choices, which could have positive long-term impacts on their health and well-being [5]. The literature highlights the importance of developing food literacy from childhood to support healthy eating habits later in life and to prepare tomorrow’s adults to become self-determined in terms of healthy eating practices [6,7].

Although many definitions have been proposed in the literature, food literacy can be defined as “food skills and practices that are learned and used across the lifespan to participate within a complex food environment. It also means considering the social, cultural, economic and physical factors related to food [8] (p. 36)”. Despite the lack of consensus regarding its definition [9], experts seem to agree that food literacy is a much more complex and multidimensional concept that extends beyond acquiring cooking skills or interpreting food labels [10]. It encompasses not only food knowledge and skills but also attitudes, culture, food systems, and the capacity to make informed choices [11]. Moreover, the broad range of food literacy’s definitions has led to the emergence of various frameworks defining this concept. For example, Vidgen and Gallegos [12] represent food literacy by eleven components distributed in four domains: food planning and management, selection, preparation, and eating. In addition, Slater [13] has proposed an organizational framework for food literacy development, illustrating this notion through a continuum of functional, relational and systems competencies.

During summer, healthy lifestyle habits tend to deteriorate among youth, which can be partially explained by the lack of structure, organized activities, and consistent adult supervision that schools normally provide [14,15,16]. As in many other countries [17], Canadian summer camps host more than 6 million youth every summer during two months (July–August) [18] and are therefore high-potential environments for promoting healthy diets among children. While food literacy is increasingly studied in the context of school settings, very little research has explored this concept in summer camps [19]. Yet these settings, much like schools, offer conditions that are highly conducive to children’s food learning [20]. They provide continuous support from camp counselors—who act as influential role models—and offer numerous opportunities for hands-on experimentation, which is aligned with cognitive and behavioral processes that facilitate the adoption of healthy eating behaviors among youth [7,21]. They also have distinctive features that set them apart from school environments, including a more playful atmosphere, greater structural flexibility, and a wider variety of activities [22]. Thus, developing a food literacy model adapted to the summer camp context could be an effective way to promote healthy eating by helping camp counselors integrate relevant initiatives throughout their daily schedules.

This qualitative study addressed the following research question: “How do camp managers, counselors, and registered dietitians (RDs) perceive the clarity, usefulness and relevance of a food literacy model adapted for Canadian summer camps?” For this purpose, this project was intended (1) to explore how camp managers and counselors perceived the clarity and usefulness of a food literacy model adapted for Canadian summer camps in order to inform its development, and (2) to explore how registered dietitians perceived the clarity and relevance of a food literacy model adapted for Canadian summer camps in order to inform its development. In addition, as a knowledge mobilization objective, this study aimed to propose a food literacy model for camp counselors, which will be further implemented in summer camps, through a collaboration with Fondation Tremplin Santé (FTS)—a non-profit organization that is dedicated to the promotion of healthy eating habits among youth, particularly in summer camps across Canada. FTS played a central role in this project as a study partner. Its contribution included sharing its preliminary work on developing a food literacy model and facilitating the selection of a variety of camps through its database, which lists hundreds of camps across Canada.

## 2. Materials and Methods

### 2.1. Study Design

**Figure 1:**
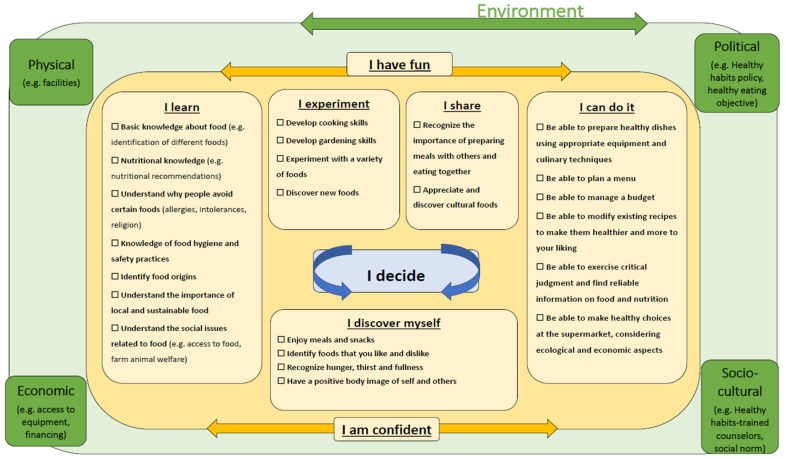
First version of the food literacy model adapted for summer camps (translated from French (original language)).

Prior to this study, FTS worked on a preliminary food literacy model based on models identified in the scientific literature [12,23,24], behavior change theories [25,26], Canadian public health policies [27,28] as well as its expertise in the field. As a result, FTS proposed 10 main components that should be included in a food literacy model adapted to the summer camp context [19]. These include food knowledge and skills (e.g., culinary abilities), relationship with food (e.g., body image), self-efficacy (e.g., applying knowledge), confidence, fun, supportive environments, behavioral theories (e.g., social norms, attitudes, perceived control), and factors influencing decision making. Finally, the last component focuses on maintaining or adopting healthy eating habits, which is the primary goal of the model. Based on this preliminary work, the first version of the adapted food literacy model used in this project was then developed, as shown in Figure 1. This first version also included more recent food literacy models, such as the Food Literacy Progression framework [13], to enrich the content of each component.

This qualitative study also used a bottom-up approach [29]. Firstly, data was collected from the field with summer camp counselors and managers to consider their practical insights regarding the first version of the model. For this study, camp counselors were either teenagers starting from 14 years old or young adults who were responsible for the supervision of children aged between 4 and 17 [30] at camp and throughout camp activities (adapted from [31]). They were supervised by camp managers who were adults involved in several duties related to the overall organization and maintenance of the camp (adapted from [31]). During this study, camp counselors and managers were asked to share their perspectives on the clarity and usefulness of the first version of the model, leading to the development of a second, field-based version of the model. Subsequently, RDs’ perspectives were solicited to ensure the scientific relevance of the second version of the model, which led to the development of a third and final version of the food literacy model. Each stage of this study, including the participant recruitment, data collection and data analysis, was carried out by the first author, with weekly support from a qualitative research expert and supervised by the research team.

### 2.2. Participants and Recruitment

In winter 2023, Canadian summer camps located in the provinces of Quebec and Ontario were contacted by email from a pre-selected contact list of FTS member camps. This collaboration facilitated the recruitment of camp counselors and managers working in a variety of summer camps that differed in terms of their locations, sizes, types, neighborhood incomes and healthy eating initiatives already implemented. The characteristics of the participating camps contributed to a greater diversity of discourses among participants, but they were not included in the data analysis. This study used a purposeful sampling strategy, more specifically, a criterion sampling as outlined by Patton [32], whereby camp counselors and managers and then RDs were required to meet specific pre-established criteria. According to the inclusion criteria, participants from summer camps were required to work as either a camp manager or camp counselor and be of at least 14 years of age. During spring 2023, the first author contacted camp managers via email to schedule an in-person visit to the camp. Subsequently, between July and August 2023, camp counselors were recruited on-site prior to the focus groups. As suggested by our partner FTS, based on its previous experiences, only camp counselors received 20$ as an incentive to participate in this study at the end of the focus group. Between October and December 2023, camp managers were recruited by email. Regarding RDs, they were recruited by email in January and February 2024 from a list of RDs from Canada proposed by the research team and FTS. The intent was to recruit at least five RDs working in the field of public health nutrition in different contexts (e.g., governmental, academic and community), as qualitative research guidelines commonly recommend samples of 5 to 30 participants and acknowledge that exploratory studies often benefit from smaller, diverse and information-rich samples [33]. According to the inclusion criteria, each dietitian recruited was required to have expertise in food literacy among youth and was also registered as a professional dietitian (i.e., with a provincial dietetic regulatory body). This study was approved by the Comité d’éthique de la recherche avec les êtres humains de l’Université Laval (CERUL) (# 2023-106 R-1/7 May 2024) through full review. Written informed consent was obtained from all participants.

### 2.3. Data Collection

In the summer of 2023, focus groups (one hour each) with camp counselors were conducted in French on-site at different Canadian summer camps. Then, between October 2023 and February 2024, individual semi-structured interviews (one hour each) with camp managers and RDs were carried out in French and online using the Microsoft Teams application. To carry out interviews and focus groups with participants, two different semi-structured interview guides were developed and pilot-tested with camp counselors, camp managers and RDs prior to data collection. The first interview guide was used for focus groups with camp counselors and was then slightly adjusted for the individual interviews with camp managers. Examples of questions asked are: To support your understanding of the model, what suggestions do you have for improving it? What are the barriers that can limit the use of this model at camp? What are the model’s strengths? (see Supplemental Table S1). A second interview guide was developed for the RD interviews and was sent to RDs with the second version of the adapted model a few days before the interview. This guide included questions such as: How does this model align with your vision of a food literacy model adapted to a summer camp context? What do you think of the relevance of this model’s constructs? (see Supplemental Table S2). Each focus group was audio recorded using a cell phone, and each individual interview was audio and video recorded using Microsoft Teams.

### 2.4. Data Analysis

After each individual interview and focus group, a summary was completed, inspired by Huberman & Miles [34]. All interviews and focus groups were transcribed manually by a research assistant and were then revised for accuracy and confidentiality by the first author and formatted for use in NVivo software with the method proposed by Bazeley [35]. Each subject was identified by a number to ensure anonymity. A qualitative thematic content analysis of transcribed verbatim was conducted using NVivo software (version 14) to identify the main themes through an inductive approach [36]. The coding tree was initially based on the main themes covered by the interview guides and discussed with the research team. The coding process was conducted during and after data collection as an iterative process [34]. Similar responses as well as relevant textual items related to the research objectives were grouped under the same code or under new codes. Codes were then grouped into broader categories, creating main themes and subthemes [37]. Three meetings (pit-stops [35]) were held with the research team at the midpoint of the codification process for the focus groups with camp counselors, and then for the interviews with managers and RDs, to discuss, clarify and improve the coding tree. Advanced analytical tools from the NVivo software were used to outline the researcher’s reflections (e.g., journaling, memo writing) and to facilitate the analysis of the data collected (e.g., word clouds).

### 2.5. Development of the Adapted Food Literacy Model

After data analysis of all discourses from individual and group interviews with camp managers and counselors, a list of modifications to be made to the first version of the adapted food literacy model was identified for the development of a second version of the adapted model. To do this, the research team selected some modifications from the interviews’ analysis, according to a set of pre-established criteria. These included salient findings (from ≥3 focus groups or individual interviews), feasibility, relevance to the field (according to the study partner FTS and qualitative data collected from the focus groups and individual interviews with camp managers and counselors) and scientific relevance (through the literature). The judgment of the analyst was also considered in this process. Through a reflective stance [38], the analyst considered the contextual nuances of the data and ensured that the selected modifications remained aligned with participants’ perspectives in relation to the research question. In addition, the same procedure was used to develop the third version of the adapted food literacy model by identifying several modifications to be made to the second version of the adapted model from the analysis of the RDs’ interviews and according to the same criteria. The third version of the adapted model was also presented and discussed with the partner FTS with the intention of ensuring its relevance within the field. All the figures illustrating the different versions of the models were created using Microsoft Word.

## 3. Results

### 3.1. Characteristics of Participants

Six Canadian French-speaking summer camps accepted to be involved in this study (see Table 1). A total of six focus groups were conducted with camp counselors (*n* = 28; *n* = 3 to 6 per summer camp), and five individual interviews were carried out with camp managers (*n* = 5). From the six camps involved, one camp manager did not take part in this study, having left the organization at the time of data collection. Furthermore, RDs working in the field of public health nutrition (*n* = 6) were recruited to be part of this study, which included two university Assistant Professors, two RDs working in governmental organizations and two RDs involved in the development of nutrition education programs for schools and communities.

### 3.2. Model Clarity Among Camp Counselors and Managers

Salient findings from participants (counselors and managers) about the clarity of the first version of the adapted model (Figure 1) were grouped into two main themes (as detailed in Table A1).

#### 3.2.1. Less Understood Elements

Five elements were identified in the participants’ discourse as less understood throughout the model. Firstly, many counselors less understood the intended audience of the model (subtheme: who will use the model?), wondering whether this model was designed to be used by children or themselves. The *Environments* section (which included *political*, *economic*, *physical* and *socio-cultural* environments at camp) was also less understood, mostly by counselors, as they indicated that they did not understand their meaning (especially regarding the *political* environment) or how to integrate them into their daily camp schedules. In addition, participants had less understanding of the model subcomponent *Be able to manage a budget*, underlining their difficulty in knowing how to implement it concretely within the context of summer camps. Some participants indicated that they may encounter challenges in applying this subcomponent among youth, particularly given that it is a responsibility more typically associated with the manager. The central part of the model (*I decide*), which represents the model goal related to the development of decision-making skills toward healthy eating, was also less understood by counselors. They expressed a lack of clarity regarding the meaning and importance of this section, pointing out that it was incomplete and imprecise. Finally, counselors lacked understanding of the significance of the arrows presented in the first version of the model.

#### 3.2.2. Ideas to Increase the Clarity of the Model

Participants proposed adding pictures in the model to enhance their understanding, grab their attention and align more closely with the playful ambience related to the camp environment.

### 3.3. Usefulness of Model According to Camp Counselors and Managers

Salient findings from counselors’ and managers’ discourses regarding the usefulness of the first version of the adapted model (Figure 1) were grouped into four main themes (as detailed in Table A1).

#### 3.3.1. The Intention to Use the Model

Participants indicated that they would use the model in their camps for three main reasons. Counselors showed an interest in using the model to help them find new activities to do with their groups of youths at camp. Moreover, some participants justified their intention to use the model to support long-term impacts on healthy lifestyles among young people. Additionally, managers indicated that they would use the model, focusing on specific model components at a time, as a guide to facilitate the development of relevant healthy eating initiatives at the camp. For instance, one manager would use the *Basic knowledge about food* (*identify different foods*) subcomponent in the model to develop a specific cooking activity.

#### 3.3.2. Barriers to Proper Use of Model

Participants indicated four types of barriers to using this model. Lack of knowledge about healthy eating among counselors was first identified as a barrier to using the model. Participants mentioned that counselors who are mainly teenagers are still in the process of developing their own food literacy, which presents a challenge in mobilizing knowledge and skills properly and with ease to youth. As another barrier to using the model, they pointed out their lack of knowledge about the usefulness of the model. Some counselors mentioned that they needed to understand why and how to use the model concretely; otherwise, they would not be tempted to use it. In addition, counselors and managers associated the lack of camp facilities, equipment, and funding to implement healthy eating initiatives as a limiting factor to using the model. For example, some counselors indicated that they were poorly equipped for cooking, which represented a barrier to organizing cooking activities with their groups of youths. Finally, the lack of motivation to use the model was also mentioned mostly by counselors, who emphasized that they were already overloaded. If the model is too complex, they will thus not be motivated to make the effort to use it.

#### 3.3.3. Ideas to Motivate Camp Counselors and Managers to Use the Model

To motivate and support the use of the model, participants suggested several ideas. They emphasized adding examples or activity ideas to each component of the model. To keep it simple, some counselors suggested that these add-ons should be separated from the model. Additionally, it was predominantly the counselors who reported that they would need training and on-site support for adequate use of the model. Some of them suggested integrating the model into the pre-camp counselors’ training at the beginning of the summer. Participants mentioned the idea of making the model more appealing by adding more fun colors and pictures and less text. They also suggested that a practical and easy-to-use model be developed and accompanied by a user guide that could include more detailed information, such as ideas for activities. They highlighted the need to provide them with opportunities to use the model, such as challenges between counselors or thematic weeks. Finally, managers also proposed simplifying the model to improve its readability for counselors.

#### 3.3.4. Adding Progression Levels to the Model

Lastly, counselors and managers agreed that adding levels of progression for each component of the model to adapt its content to age groups of young people would improve their use of the model by providing more guidance.

### 3.4. Development of the Second Version of the Adapted Model

**Figure 2:**
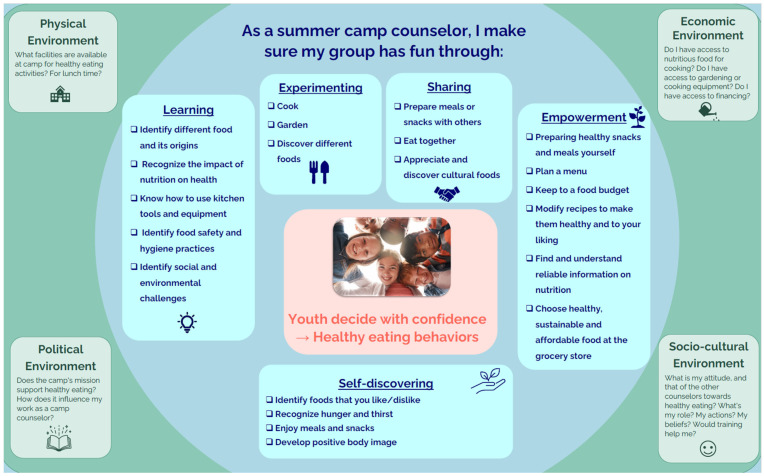
Second version of the food literacy model adapted for summer camps (translated from French (original language)).

Following the analysis of the focus groups with camp counselors and individual interviews with camp managers, and based on our criteria, several specific modifications were identified, leading to the development of the second version of the adapted food literacy model (Figure 2). Regarding the main changes compared to the first version of the model, we clarified the model’s intended audience by adding the following sentence: As a summer camp counselor, I make sure my group has fun through. We also refined the central part of the model, formerly *I decide*, by providing additional details on its overarching purpose. To make the *Environments* section clearer and more engaging for camp counselors, we incorporated specific guiding questions. We simplified the component *To manage a food budget* by renaming it *To keep a food budget*. We removed the arrows associated with the *Fun* and *Confidence* components and redistributed their contents throughout the model. We enhanced the model’s visual appeal by integrating images and colors. Finally, we grouped certain components together to simplify the model.

### 3.5. Model Clarity Among RDs

Salient findings from RDs’ discourses about the clarity of the second version of the adapted model (Figure 2) were grouped into three main themes (as detailed in Table A2).

#### 3.5.1. A Clear Understanding of the Model’s Constructs

RDs showed a clear understanding of the model, underlining that its constructs were consistent with their knowledge about food literacy. Among them, some pointed out that they recognized familiar concepts in the presented model, including food knowledge and skills, as well as the influence of the environment, which are commonly found in other food literacy and public health models.

#### 3.5.2. Less Understood Elements

Subthemes indicated that three elements were less understood. RDs reported a lack of clarity regarding the *Learning* component, especially the *Identify social and environmental challenges* subcomponent of the model, explaining that it implies overly broad notions. They also lacked understanding of the *Empowerment* component of the model, explaining that they were confused as to whether this component referred to actions to be taken at home or at camp. Lastly, the model’s visual design was an element less understood by RDs. For instance, some of them indicated that the model structure based on three levels was less clear.

#### 3.5.3. Ideas to Increase the Clarity of the Model

RDs proposed clarifying the *Empowerment* component especially by specifying that it refers to activities and actions taken at camp. In addition, they suggested modifying the *Self-discovering* component especially through the integration of more concrete and precise points to facilitate its use in the field. They proposed making a few visual changes to the model, for instance by using the same circular shape to represent the model’s three levels or by suggesting a more linear structure that could facilitate its reading. Finally, RDs emphasized the importance of the given explanations to enhance their comprehension of the model.

### 3.6. Relevance of the Model from RDs’ Perspectives

Salient findings from the RDs’ point of view regarding the relevance of the second version of the adapted model (Figure 2) were grouped into three main themes (as detailed in Table A2).

#### 3.6.1. Model’s Less Relevant Constructs

Some RDs indicated that the *Empowerment* component, especially its subcomponents *Plan a menu* and *Keep to a food budget*, were less relevant. They mentioned that they were too complex for children, less applicable to their everyday life and less adapted to the ludic context of summer camps.

#### 3.6.2. Model’s Most Relevant Constructs

In contrast, other RDs mentioned the opposite, i.e., that the *Empowerment* component, including the subcomponents *Plan a menu* and *Keep to a food budget*, was relevant. Some RDs showed a certain ambivalence about the relevance of the *Empowerment* component, underlining that it may be relevant only if introduced in a fun way and adapted to young people’s age groups. RDs who found it relevant did not agree that this component was overly complex for young people. Conversely, they highlighted that it represented an opportunity to cultivate awareness of empowerment actions, which should be initiated at an early age. This also underlines the importance of combining the recreational and educational aspects of the camps, as the playful context is central to their activities. This demonstrates that the development of more complex dimensions of food literacy, such as empowerment, food justice and sustainability among youth, can indeed occur within a playful environment.

In addition, RDs indicated that the presence of the *Environments* section was relevant and complementary to the model. Furthermore, they found it relevant and appropriate to include activity ideas in a user manual along with the model. They said that the model was comprehensive and covered relevant topics. They appreciated the central part of the model (i.e., *Youth decide with confidence* → *Healthy eating behaviors*), as well as the overall model structure. Finally, RDs agreed with the relevance of the *Learning* component, in particular the *Identify social and environmental challenges* subcomponent, underlining the importance of fostering reflections on these issues among young people. RDs agreed that the *Self-discovering* component was relevant because of the topics it covered, as well as its subcomponents, which were easy to integrate into daily camp life.

#### 3.6.3. Ideas to Improve the Relevance of the Model’s Constructs

To improve the relevance of the model, RDs suggested clarifying the *Identify social and environmental challenges* subcomponent of the *Learning* component in a more precise and concrete way. They also proposed adapting and simplifying the *Empowerment* component to make it more applicable and suitable for youth.

### 3.7. Final Version of the Adapted Food Literacy Model

After the analysis of all individual interviews with RDs, and based on our criteria, specific modifications were identified, which resulted in the development of the final version of the adapted food literacy model in the context of this study. Regarding the main changes, we clarified that the *Empowerment* component refers to activities taking place at camp by specifying this in the following sentence: As a summer camp counselor, I create opportunities at camp for my group of youths to have fun through. We also repositioned the *Empowerment* component below the other components to emphasize that it differs from them because of the greater complexity of its content. Modifications were made to the visual design to ensure that the three levels of the model are clearly represented. We further clarified and simplified the contents of certain components, such as the *Learning* and *Self-discovery* components, to make them more concrete and applicable for youth. For example, the component *To plan a menu* was revised and replaced with the component *To give recipe ideas for a menu*.

**Figure 3:**
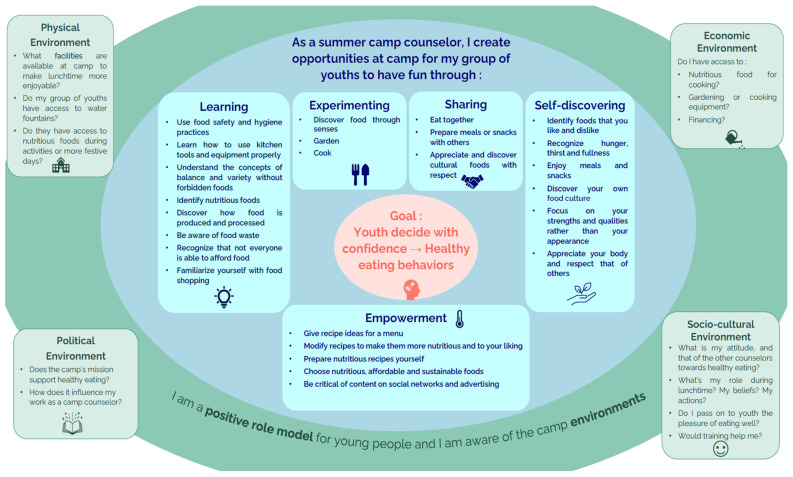
Final version of the food literacy model adapted for summer camps (translated from French (original language)).

As shown in Figure 3, the final model is structured around three circular levels, as follows: Firstly, the goal of the model (*Youth decide with confidence* → *Healthy eating behaviors*) is centered on young people, empowering them to make informed food choices that lead to the adoption of healthy eating behaviors. Then, through this model, camp counselors are encouraged to create opportunities at camp for their groups of youths so that they can have fun through five components: *Learning*, *Experimenting*, *Sharing*, *Self-discovering* and *Empowerment*. Each component of the model provides ideas to inspire counselors to implement healthy eating initiatives at their camps. Finally, the model presents camp environments such as the *physical*, *political*, *economic* and *socio-cultural* and emphasizes the need to be aware of them. It recognizes the positive influence that counselors can have on youth as role models. The *Environments* section will also help counselors to make relevant decisions that support appropriate healthy eating interventions.

## 4. Discussion

To the best of our knowledge, this is the first study to propose a food literacy model tailored to the summer camp context by exploring the perspectives of field experts, as camp managers and camp counselors, and food literacy experts, as RDs. The main findings showed that camp managers and counselors were interested in using a key-in-hand, simple and clear food literacy model, while they mentioned their need for support by incorporating practical activity suggestions, training and on-site support to facilitate its use. In addition, RDs underlined that the model generally covered all relevant and essential themes of food literacy but that there was a need to clarify specific model components and subcomponents to make them easier to understand, more concrete for counselors and more suitable for youth at camp. In the end, the final food literacy model adapted to summer camps is centered on young people and features five main components—*Learning*, *Experimenting*, *Sharing*, *Self-discovering* and *Empowerment*—while also considering the physical, economic, political and socio-cultural environments.

Since its development, food literacy has not been thoroughly explored in the context of summer camps; thus, studies are lacking to compare our findings. However, there are similarities between our findings and those from implementation studies on healthy eating programs in schools for children. In line with our findings, the main barriers perceived by schoolteachers to delivering nutrition education appropriately in elementary schools include their lack of nutrition knowledge and resources [42]. The need for training as well as access to easy-to-use teaching tools (e.g., lesson plans, interactive activities, games) have been mentioned to facilitate the delivery of these programs [42]. Given that camp counselors are usually teenagers, their food literacy is also still in development. The lack of nutrition knowledge can therefore be a significant barrier to the effective use of the model. This highlights the necessity of adequate support and resources to ensure its proper use in camps to improve children’s food literacy. Moreover, similar barriers have been noted among teachers, such as a lack of equipment (e.g., kitchen tools) and funds (e.g., to purchase cooking ingredients) to implement healthy eating activities [43,44,45], which are also consistent with our findings and reflect interesting parallels between school and summer camp settings. Overall, these barriers could represent potential challenges related to the model’s application across different summer camp contexts. Future work could include validating the model with camp counselors and evaluating its implementation in the field, which would provide a more comprehensive understanding of its practical utility and real-world obstacles.

While many components of the food literacy model developed in this study are derived from the literature, we examined similarities with other frameworks designed to develop food literacy among children. The concept of food and nutrition knowledge, included in the *Learning* component, is frequently found in food literacy frameworks for both adults and youth [5,46,47], as this notion is commonly used to define food literacy [11]. In addition, the concepts of social justice, food environmental sustainability and financial literacy have been integrated into a particular framework developed by a home economics secondary school teacher, Eric Schofield, that is used to improve food literacy among youth more specifically in high school settings [48]. These concepts were also translated into the latest version of the proposed model in the *Learning* component through the following subcomponents: *Recognize that not everyone is able to afford food*, *Be aware of food waste* and *Familiarize yourself with food shopping*.

Many food literacy interventions in elementary schools have focused on the interactive food literacy dimensions involving cooking, gardening and tasting new foods [49]. Accordingly, our food literacy model also emphasizes these interactive activities through the *Experimenting* component. As school settings, summer camps represent relevant environments for young people to engage in experimental activities related to food, which is consistent with the playful and dynamic context of summer camps [19].

The contents of the *Sharing* and *Self-discovering* components in the present food literacy model are derived from the relational competencies proposed by Slater [13]. Summer camps offer many opportunities for counselors to easily integrate these components with their groups of youths, such as simply having lunchtime where all young people eat together. Thus, *Eat together* (*Sharing*), *Enjoy meals* or *Recognize hunger*, *thirst and fullness* (*Self-discovering*) are, for instance, actions that can be readily accomplished during this shared moment.

Some food literacy models for youth include a critical dimension related to decision-making abilities and more advanced cognitive skills needed as media literacy [6,46]. The *Empowerment* component in the food literacy model developed in this study addresses these concepts through its different subcomponents, for instance, *Choose nutritious*, *affordable and sustainable foods* or *Be critical of content on social networks and advertising*. While scientific evidence supports these subcomponents [12,13,46], RDs expressed divergent opinions on their relevance, such as managing a food budget. Counselors and managers also reported their difficulty in integrating it concretely in the field. Therefore, this could indicate a gap in applying scientific knowledge on the ground with those involved.

In addition, many studies have incorporated broader environmental influences underlying food systems, the social context and the community level into their definitions of food literacy and frameworks [5,6,11,47,50]. Environmental influences were also incorporated into the current food literacy model through the presence of different types of *Environments* related to summer camps that support youth’s development [22,51,52].

This study shows many strengths, as it is among the first studies to explore the development of food literacy in the context of summer camps involving summer camp stakeholders and RDs. Our findings were summarized through the development of second and third versions of the model, which brought an added value, as we co-created with participants a relevant and adapted model that is understandable and usable in summer camps. The collaboration with FTS also represented a strength of this study through its expertise and role in knowledge mobilization. Data triangulation using multiple data sources [53] was achieved by collecting data from two types of participants at each camp, camp counselors and managers, which resulted in the emergence of two complementary visions of the understanding and usefulness of the model. In addition, a preliminary literature review was combined with RDs’ interviews to provide a more comprehensive understanding of the accuracy of the food literacy model constructs.

## 5. Limitations

Although innovative, this exploratory study has some limitations, such as its small sample of participants, which may limit the transferability of the findings to other populations and settings. The reduced sample size is partly explained by the small and fluctuating number of participants in the focus groups (between three and six), which reflected the varying sizes of the camps involved. For instance, one camp had only three counselors, which necessarily restricted the size of its focus group. Participants were recruited from a specific cultural and geographic context, which may not reflect the perspectives of professionals working in different educational or socioeconomic environments. Thus, the findings do not represent the perspectives of all counselors, managers and RDs in the provinces of Quebec and Ontario, and the final food literacy model cannot be transferred to all summer camps in Canada. In addition, this study relied primarily on subjective perceptions from camp stakeholders, without a direct evaluation of youth participants or an objective assessment of food literacy outcomes. The proposed framework was not experimentally implemented or validated through intervention studies, making it difficult to determine its practical effectiveness in improving dietary behaviors, food autonomy or nutritional knowledge. Further steps could therefore include validation of these findings with a larger sample of participants and evaluation of the model’s implementation and effectiveness in the field to enhance the transferability of the results.

Due to limited time and resources, the first author was the only coder to perform data analysis, supported by the research team. For the same reasons, data saturation was not reached; however, this was not the objective, as this was an exploratory study. However, during the data collection and analysis, the number of new codes related to the research objectives decreased, which suggested that we were approaching inductive thematic saturation [54]. Potential researcher interpretation bias inherent to thematic qualitative analysis should also be considered, even though the analyst employed a reflective approach throughout this study. All managers and counselors involved in this study were part of an FTS member camp and may have already been interested in or aware of healthy eating, which may suggest selection bias that can be transposed into the findings [55]. We tried to reduce this bias by considering the camps’ previous experiences of healthy eating while selecting them. Finally, factors that extended beyond the camp environment, such as family influence, economic barriers, and cultural diversity, as well as the long-term sustainability of the proposed educational strategies, were not deeply explored.

## 6. Conclusions

This study contributes to the growing field of food literacy by proposing an adapted framework designed for summer camp counselors. The findings suggest that food literacy in children and adolescents extends beyond nutritional knowledge and includes social, environmental, behavioral, and empowerment dimensions that may influence food-related decision making. The qualitative perspectives obtained from camp managers, counselors, and dietitians highlight the importance of creating supportive and interactive food environments that encourage autonomy, critical thinking, and healthy eating behaviors among youth. Furthermore, the proposed framework also emphasizes the relevance of experiential learning and age-appropriate educational strategies in promoting food-related competencies. However, the model should be interpreted as an exploratory framework rather than a validated intervention tool. Additional empirical research is necessary to determine its applicability, reproducibility, and long-term effectiveness in different socio-cultural and educational contexts. Overall, this study reinforces the potential of summer camps as complementary environments for food and nutrition education and highlights the importance of integrating food literacy into broader health promotion initiatives targeting young populations.

## 7. Perspectives

Future studies should focus on validating and testing the proposed food literacy model in real-world summer camp interventions involving diverse youth populations. Longitudinal and mixed-methods studies are needed to evaluate whether the framework effectively improves food knowledge, dietary behaviors, autonomy, and long-term health outcomes. Further research should also investigate how socio-cultural, economic, and family-related factors influence the development of food literacy among children and adolescents. In addition, future interventions may benefit from integrating digital educational tools, interactive learning strategies, and family participation to strengthen food-related competencies beyond camp settings. Comparative studies across different countries and educational environments could help establish the broader applicability and cultural adaptability of the framework. Incorporating quantitative indicators and validated assessment instruments may contribute to improving the scientific robustness and translational relevance of food literacy research.

Beyond the context of summer camps, this model could potentially be applied by youth organizations with similar settings for involving children in food literacy activities. It could also inspire nutrition education practitioners to advance research on food literacy among youth from the perspective of developing relevant strategies and policies. Thus, this model will contribute to mobilizing youth stakeholders towards relevant healthy eating actions that will ultimately have positive impacts on children’s health and well-being.

## Figures and Tables

**Table 1:** Socioeconomic and demographic characteristics of participating summer camps (*n* = 6).

Camp	Province	Localization ^a^	Size ^b^	Type	Neighborhood Income ^c^	Active Member of FTS ^d^	*n* Counselors Recruited (*n* Total 28)	*n* Managers Recruited (*n* Total 5)
1	Québec	Urban	Large	Day camp	Advantaged	Yes	4	1
2	Québec	Rural	Large	Day/ overnight camp	Disadvantaged	Yes	6	1
3	Ontario	Urban	Large	Day camp	Disadvantaged	No	5	1
4	Québec	Rural	Large	Family camp ^e^	Disadvantaged	No	4	-
5	Québec	Rural	Small	Day camp	Disadvantaged	Yes	3	1
6	Québec	Urban	Medium	Day camp	Disadvantaged	Yes	6	1

^a^ According to the postal code of camps from the FTS database and the identification of rural/urban areas proposed by INSPQ [39] and Statistics Canada [40]. ^b^ Camp size is categorized according to the number of young people in the camp: small (≤50 children), medium (between 50 and 100 children) and large (≥100 children). ^c^ Based on the FTS disadvantage index according to its database. ^d^ As FTS member camps, they have access to training sessions, tools, contests, and challenge-based activities related to healthy eating and are monitored by FTS through its database. ^e^ Family camps host children, especially those with special needs, and their families in an inclusive and accessible setting [41].

## Data Availability

The original contributions presented in this study are included in the article/Supplementary Materials. Further inquiries can be directed to the corresponding author.

## Supplementary Materials

The following supporting information can be downloaded at: https://www.mdpi.com/article/10.3390/nu18132168/s1, Table S1: Semi-structured interview guide with camp counselors and managers; Table S2: Semi-structured interview guide with registered dietitians.

## Abbreviations

The following abbreviations are used in this manuscript:

RDs	Registered dietitians
FTS	Fondation Tremplin Santé

## Appendix A

**Table A1:** Main and subthemes related to clarity and usefulness of the model as perceived by camp counselors and managers, in decreasing order of salience (*n* = 33; six focus groups, five individual interviews).

Overarching Theme	Main Theme	Subtheme	Frequency ^a^		Illustrative Example ^b^
			*n* Focus Groups	*n* Individual Interviews	
Model clarity among camp counselors and managers	Less understood elements	Who will use model?	5	1	“Well, in a way that’s more understandable for children, because when you show them this [the model], I don’t think they’ll understand.” (Counselor 01, camp 06) “No, but it’s [the model] for us.” (Counselor 06, camp 06)
		*Environments* section	5	1	“Well, it seems that what’s in yellow in the middle attracts me more and I find it more interesting than what’s in green [*Environments* section] around it. What’s in green around it looks like… I understand it a little less, like yeah.” (Counselor 01, camp 01)
		*Be able to manage a budget* model subcomponent ^c^	3	2	“Well, uh it’s not up to us as often we have to talk to like our supervisors like uh X, often it’s X, he’s the one in charge of that [to manage a budget]. And like, yeah, it’s really unclear.” (Counselor 04, camp 03)
		Presence of arrows in model ^c^	4	0	“I don’t understand arrows so well, I think. Where do the arrows go, what do they do?” (Counselor 02, camp 01)
		Central part of model (*I decide*) ^c^	3	0	“I think everyone is asking the question what do *I decide*, am I the only one?” (Counselor01, camp 06)
	Ideas to increase the clarity of the model	Add pictures	3	2	“Words are like confusing, it’s scary, if you had like lots of pictures, I’d be more attracted to look and like understand [the model] (…) When I see a lot of letters like that, it just, it stresses me out it reminds me of school I’m like no.” (Counselor 02, camp 03)
Usefulness of model according to camp counselors and managers	Intention to use the model	For new activity ideas	4	0	“I find that yes, it [the model] can be useful because I find that, uh especially when we have new camp counselors (…) because every day we try to find new ideas for activities and we try to plan our weeks, but it seems that there is not much to do (…)” (Counselor 01, camp 03)
		For long-term impacts on healthy lifestyle habits	3	2	“Well, because I think it’s important for children to learn to eat well at an early age, and to understand the importance of a healthy diet. (…) the younger you learn it, the better it will be for your future development.” (Counselor 01, camp 05)
		As guide to focus on specific model components to develop activities at camp	0	3	“(…) I think that the way [the model] it’s set up, we could really focus more on one of the circles [one model component] to develop a specific activity (…)” (Manager, camp 01)
	Barriers to proper use of model	Lack of knowledge among counselors about healthy eating	2	3	“There may even be counselors who’ve never cooked before in their lives. You know, that’s for sure, that’s for sure. And now we’re asking them to lead something they’ve never experienced themselves, so it’s a bit special.” (Manager 01, camp 01)
		Lack of knowledge among counselors about usefulness of model	3	2	And sometimes, they [camp counselors] don’t see the importance of that [the model], and they might say, “Oh, what’s this thing for,” like “I don’t want to use it,” hum they don’t see why like they should use it. (Counselor01, camp 01)
		Lack of facilities, equipment and funding for healthy eating activities	3	2	“As for funding, well, we’ve got nothing. Except from picnic tables outside and, uh, hoping to get what we need in the kitchen, we don’t have much. In any case, we’re talking about here because we’re a non-profit organization, so maybe other summer camps have a bigger budget and more resources (…)” (Counselor01, camp 04)
		Lack of motivation among counselors	3	1	“(…) if it’s left like that [the model] to counselors, um, I know quite a few counselors who wouldn’t want to use it, they’d be like I don’t want to use it, I’m not motivated to do that, I’ll just do something I usually do (…)” (Counselor 03, camp 02)
	Ideas to motivate camp counselors and managers to use the model	Add activity ideas	5	5	“It’s much easier to follow a model like that when you know what you’re doing in practice. We’re going to, we’re talking about creativity because when we have that [the model], we create the activity on the spot and if the activity’s already there, we just put into practice everything that’s there it’s easier.” (Counselor 02, camp 03)
		Need for training	4	2	“I know it’s not easy, but integrating it [the model] into training for camp counselors, so that they see it like in a training and, to have the diagram [the model] and make all this like a bit interactive. That would be it, it’s really going to get more into the heads of the, of the counselors I think.” (Counselor 02, camp 01)
		Make model more appealing (colors, less text, pictures)	3	2	“Well, I think it has to be colorful, it has to be, as I said, not too overloaded, not too, uh, with simple, simple words, some, a little descriptive but not too much. And it’s got to be visually appealing uh yes the colors, but it’s got to have, like, a youthful look too (…)” (Manager, camp 05)
		Need for trained resource person in field	4	0	“And at day camp, we always have resource people, like camp leaders. So, it’s also up to them, I think uh, to explain and to train if there are any questions it’s up to them to answer.” (Counselor 04, camp 06)
		Practical model with user guide	3	1	“(…) one thing that can be done is, to have both in the sense of having a very simple and practical model like this, which is easy to use and, for people who need more, well, to provide a, a reference manual or a reference guide available (…)” (Counselor03, camp 02)
		Provide opportunities for counselors to use model	3	1	“It’s… Well, it depends on each camp, but I know that… There’s a lot of competition among the, um, counselors. And, often, games between them work a lot like “Hey, at least once in your week it’d be cool if you used this wonderful tool [the model], do a really cool activity”, and then you earn points for your team or whatever. Usually it really motivates the counselors to surpass themselves (…)” (Counselor 05, camp 02)
		Simplify model	0	3	“(…) I think you have to make it [the model] as practical as possible, as cute, as, uh, colorful, with as little text as possible, because not to be judgemental, but the generations we have are reading less and less. So, it’s got to be simple.” (Manager, camp 05)
	Add progression levels to the model	Facilitate use of model by providing more guidance for camps	3	2	“Well, I think it’s a good idea [adding levels of progression in the model] (…) well, if you have levels, then you can say to yourself, okay, well, I’m looking at this. It reminds you to look more often at the model and to say “okay level one is done”. Then, you move on to the next one.” (Counselor 003, camp 05)

^a^ Only salient findings from ≥3 focus groups or interviews are included in this table. ^b^ Quotations have been translated from French (original language) to English. ^c^ Refers to components and sections identified only in the first version of the adapted food literacy model.

**Table A2:** Main and subthemes related to clarity and relevance of the model as perceived by registered dietitians (RDs), in decreasing order of salience (*n* = 6).

Overarching Theme	Main Theme	Subtheme	Frequency ^a^ (*n* Individual Interviews)	Illustrative Example ^b^
Model clarity among RDs	Clear understanding of the model’s constructs	In line with dietitian knowledge of food literacy	4	“(…) we really do find all the components related to knowledge and skills, umm, that we find in other food literacy models, I find it really comprehensive with the environments, the way it’s built.” (Dietitian 04)
	Less understood elements	*Learning* component	3	“And I found *Identify social and environmental challenges* [sub-component of the *Learning* component] to be really vague. (…) I’d be curious to ask a camp counselor to name some social and environmental challenges. I’d be curious to see what the answer would be, um, even for me, what would I say to that? It’s so broad as a question that I wasn’t sure what the objective was.” (Dietitian 01)
		*Empowerment* component	3	“(…) Let’s say I look at the *Empowerment* [component] and I ask myself, Is this something that should be done at camp, or, will it really to help young people develop their autonomy, in all aspects of their lives?” (Dietitian 02)
		Model visual design	3	“(…) I still had questions about my understanding, especially the pink bubble with the young person in the center. (…) because of the shape of the pink bubble, I was under the impression that it was part of the bigger blue bubble. And I couldn’t understand why it was there.” (Dietitian 01)
	Ideas to increase the clarity of the model	Clarify *Empowerment* component	3	“When I read that aspect [*Empowerment* component], I see the young person outside of camp. If you want it to be cooking at camp, you have to specify that. You know, preparing your own snacks and meals or making simple recipes, in the context of camp or culinary activities. Because that’s not the same thing at all.” (Dietitian 01)
		Modify *Self-discovering* component	3	“Well, actually, the whole Self-discovering part, body image, I’ve still got my little star of, you know, whatever that means, so maybe it’s worth going into more detail.” (Dietitian 03)
		Modify visual design of model	3	“I’m almost wondering if, and I liked the idea of putting the youth at the center of the model, but I’m almost wondering if we should put the youth at the top, and then it’s like, as a camp counselor, how am I going to support my youths towards this model goal in pink? And then, you put *Learning*, *Experimenting*, *Sharing*, *Self-discovering* and a bit of the *Empowerment* at the bottom.” (Dietitian 02)
		Need explanations of the model to better understand it	3	“On the other hand, I’m glad you explained it [the model] to me because I think that if, well, I had just skimmed over it, for example the *Empowerment* circle, I’m not sure I would have understood it the way you explained it to me.” (Dietitian 02)
Relevance of the model from RDs’ perspectives	Model’s less relevant constructs	*Empowerment* component	3	“There are really a lot of components, I think, before integrating *Plan a menu* [sub-component of the *Empowerment* component], you know, I think it’s much more important to talk about balanced eating so that young people are able to say, Okay, I’ll prioritize, I’ll get a piece of fruit instead of a cookie as a snack, because for such and such a reason. I think that’s a choice that’s more at his or her level.” (Dietitian 01)
	Model’s most relevant constructs	*Empowerment* component	5	“For example to *Plan a menu* [sub-component of the *Empowerment* component], it’s good that they are practicing, obviously it’s not their responsibility to plan the family’s menu. But I think it’s interesting to set it out in advance and say, well, maybe there’s an activity related to this or that would be cool (…)” (Dietitian 02)
		*Environments* section	5	“I like the *Environments* with the different circles in green. I find that it brings this nuance to food literacy to talk about environments and the different types of environments, because I thought they were well constructed, well presented and clear.” (Dietitian 02)
		Accompany model with user manual of activity ideas	4	“But you answered my question by saying that there would be a user manual with lots of ideas for activities, that’s what they need, something concrete to actually implement it [the model] in camps (…)” (Dietitian 04)
		Complete model addressing relevant themes	4	“(…) I think you’ve named social and environmental challenges, culture, etc., which are often the things I find missing in food literacy models, but you have it.” (Dietitian 02)
		Central part of model (*Youth decides with confidence* → *Healthy eating behaviors*) ^c^	4	“(…) Well, it’s obvious that putting the *Youth decides with confidence*, at the center of the model, is something we have to keep in my opinion.”
		Model structure	4	“The circles, the center, the *Youth* in the center of the model, that’s totally logical, the circles around it, I think it represents really well, I saw really quickly like that, the, the main points. And the environments to support it.” (Dietitian 03)
		*Learning* component	3	“But I was happy to see it there [*Identify social and environmental challenges* model sub-component of the *Learning* component], and I think it’s very appropriate, even for four- and five-year-olds, and yes, there are ways of doing it that respect age and intellectual development, but I think it’s a must, and it has to be there.” (Dietitian 02)
		*Self-discovering* component	3	“Uh, I’d go with the daily thing, which for me is, well, getting to know ourselves better in terms of taste, hunger cues and feeling full (…) Discovery, I think that’s also important, because it includes really playful aspects, but uh, discovery combines really well the mission of the camp and food.” (Dietitian 03)
	Ideas to improve the relevance of the model’s constructs	Clarify *Identify* *social and environmental challenges* subcomponent ^c^ of *Learning* component	3	“So, this one [*Identify social and environmental challenges* sub-component of the *Learning* component] bugs me, I think there’s a way to modify it so that it’s clearer, and by really asking the question, what do I really want the youth to do actually?” (Dietitian 01)
		Adapt certain subcomponents of *Empowerment* component to make them more applicable to youth	3	“(…) as long as children are at home, they participate and make suggestions, but it’s the role, it’s the parent who will ultimately make the menu, it’s not the child so if you want to add a menu component [related to the *Plan a menu* sub-component of the *Empowerment* component], it would be more to say, once again, let’s be able to give ideas for a menu, let’s suggest recipe ideas let’s say for the week’s menu to his or her parents (…) I always ask myself the question, what is the youth going to do? What’s gonna have the most impact on him or her tomorrow morning.” (Dietitian 01)

^a^ Only salient findings from ≥3 interviews are included in this table. ^b^ Quotations have been translated from French (original language) to English. ^c^ Refers to components and sections identified only in the second version of the adapted food literacy model.
