# Supplementary material for: Designing a Model for Developing Food Literacy Among Youth: Insights from Summer Camps
Source: Nutrients. 2026 Jul 3;18(13):2168. doi: 10.3390/nu18132168 (PMC13363836; doi:10.3390/nu18132168)
Supplement: Supplementary file 1 [file nutrients-18-02168-s001.zip › nutrients-4320568-supplementary.pdf]

Supplementary material

Manuscript: "Development of a model for improving food literacy in summer camps: a qualitative study"

Supplemental Table S1. Semi-structured interview guide with camp counselors and managers

Introduction questions<br>Brief presentation of the **first version** of the adapted food literacy model	
**Explore the clarity of the model**	• *To support your understanding of the model, what suggestions do you have for improving it?*
**Explore the usefulness of the model**	• *When you see a model like this, would you use it in your camp? For what reasons?*<br>• *Do you think it might be useful to identify levels of progression for each component of the model (e.g. for Basic knowledge about food subcomponent, level 1 would be being able to identify different foods, level 2 would be knowing food preservation methods, etc.)?*<br>• *What are the barriers that can limit the use of this model at camp?*
**Overall model improvements**	• *What are the model's strengths?*<br>• *Do you have any other suggestions for improving this model?*
Ending questions	

Supplemental Table S2: Semi-structured interview guide with registered dietitians

Introduction questions<br>Brief presentation of the **second version** of the adapted food literacy model	
**Explore the clarity of the model**	• *How the model constructs seem to be in adequacy with your knowledge on the subject? For what reasons?*<br>• *How would you improve this model to enhance understanding?*<br>• *How does this model align with your vision of a food literacy model adapted to a summer camp context? For what reasons?*
**Explore the relevance of the model's constructs**	• *What do you think of the relevance of this model's constructs?*<br>• *Are there any components of the model that you feel are less relevant and could be removed? If so, which ones and why?*<br>• *Which components of this model are the most relevant to keep?*<br>• *Are there any missing components in this model? If so, which ones and for what reasons?*<br>• *What other improvements would you make to this model to ensure its relevance and accuracy?*
Ending questions	
